# Supplementary material for: Resting-State Theta Oscillations and Reward Sensitivity in Risk Taking
Source: Front Neurosci. 2021 Apr 28;15:608699. doi: 10.3389/fnins.2021.608699 (PMC8113640; doi:10.3389/fnins.2021.608699)
Supplement: Supplementary file 1 [file Data_Sheet_1.PDF]

frontiers

Supplementary Material

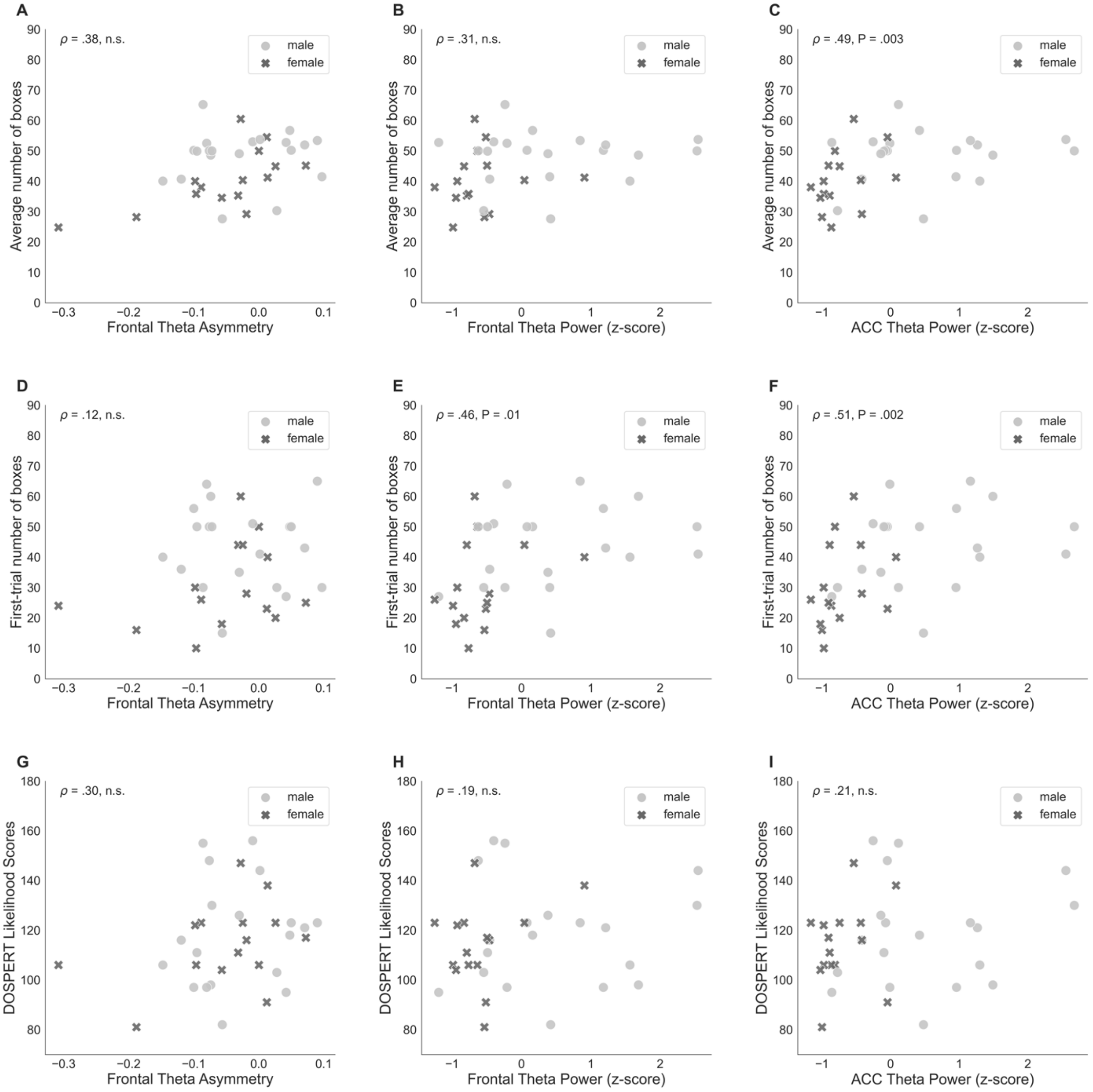

Supplementary Figure 1. Spearman's correlations of risk-taking measures and neurobiological measures based on resting-state recordings before the game. P-values are reported only for correlations significant after FDR-control. DOSPERT - Domain-Specific Risk-Taking Scale. ACC - anterior cingulate cortex.

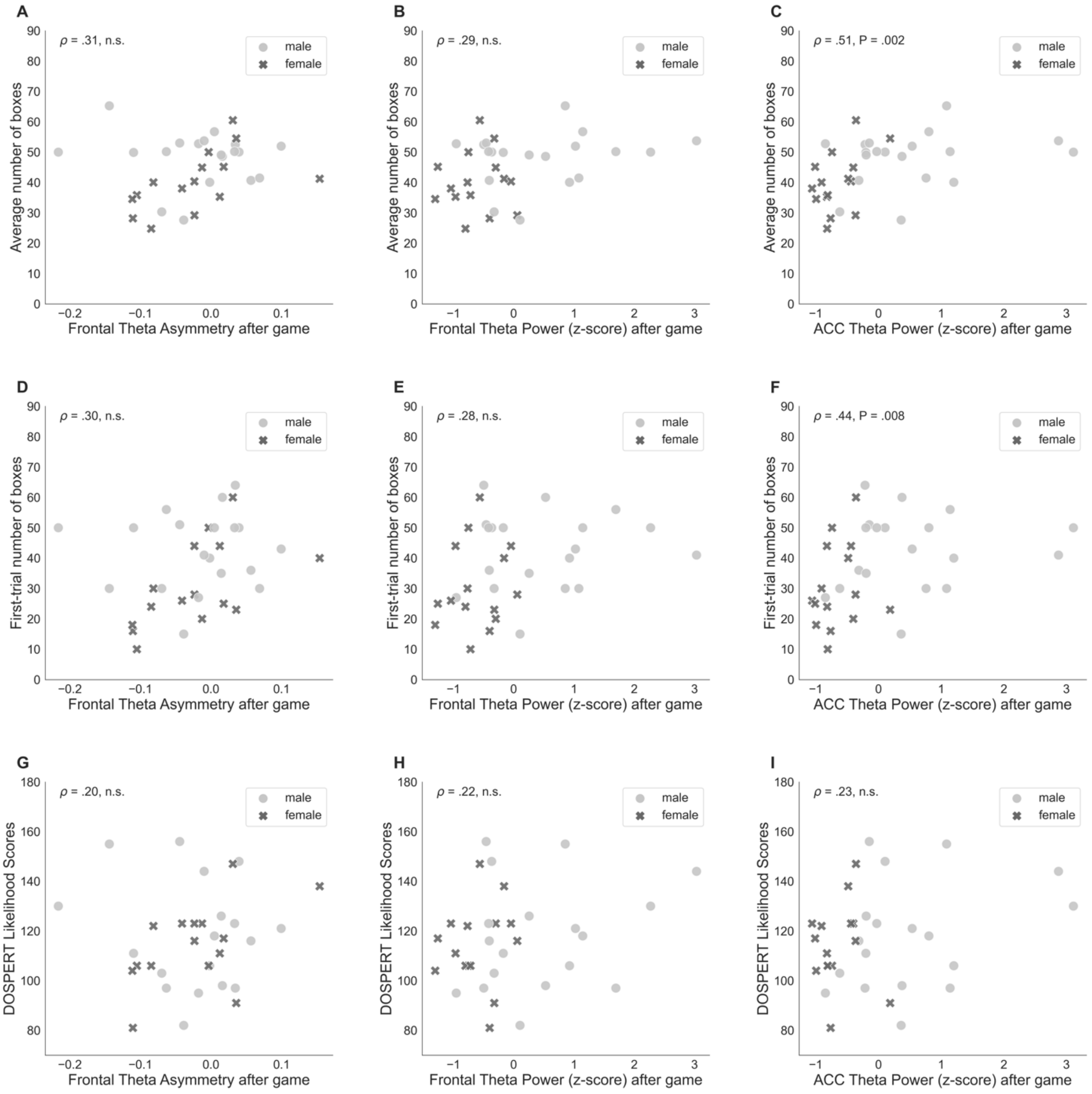

Supplementary Figure 2. Spearman's correlations of risk-taking measures and neurobiological measures based on resting-state recordings after the game. P-values are reported only for correlations significant after FDR-control. DOSPERT - Domain-Specific Risk-Taking Scale. ACC - anterior cingulate cortex. Females and males did not differ significantly in rsFTA after the game (Mann-Whitney U-test P = 0.47). Males had significantly higher rsFT (Mann-Whitney U-test P = 0.0006) and ACC theta power (Mann-Whitney U-test P = 7.68e-06) after the game than females. rsFTA before and after the game showed a significant, but moderate non-parametric association (Spearman's ρ = 0.47, P = 0.004, FDR-controlled), while rsFT before and after the game correlated more strongly (Spearman's ρ = 0.85, P = 2.73e-10, FDR-controlled). Finally, ACC theta power before and after the game highly and significantly correlated as well (Spearman's ρ = 0.94, P = 1.19e-16, FDR-controlled).

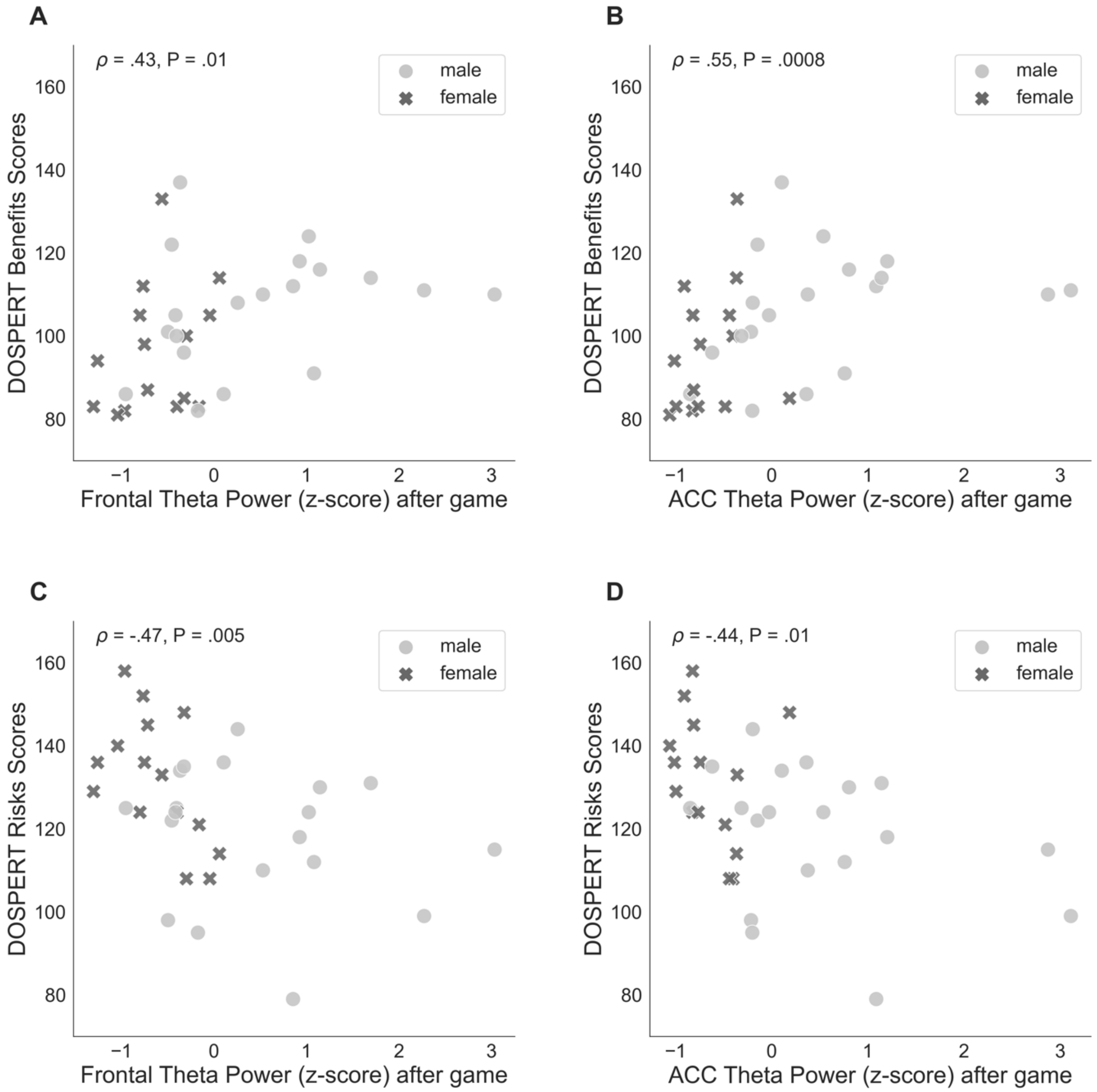

Supplementary Figure 3. Spearman's correlations of self-assessed measures of reward (DOSPERT benefits) and punishment (DOSPERT risks) sensitivities with frontal theta power and ACC theta power, standardized. P-values are reported only for correlations significant after FDR-control. DOSPERT - Domain-Specific Risk-Taking Scale. ACC - anterior cingulate cortex.

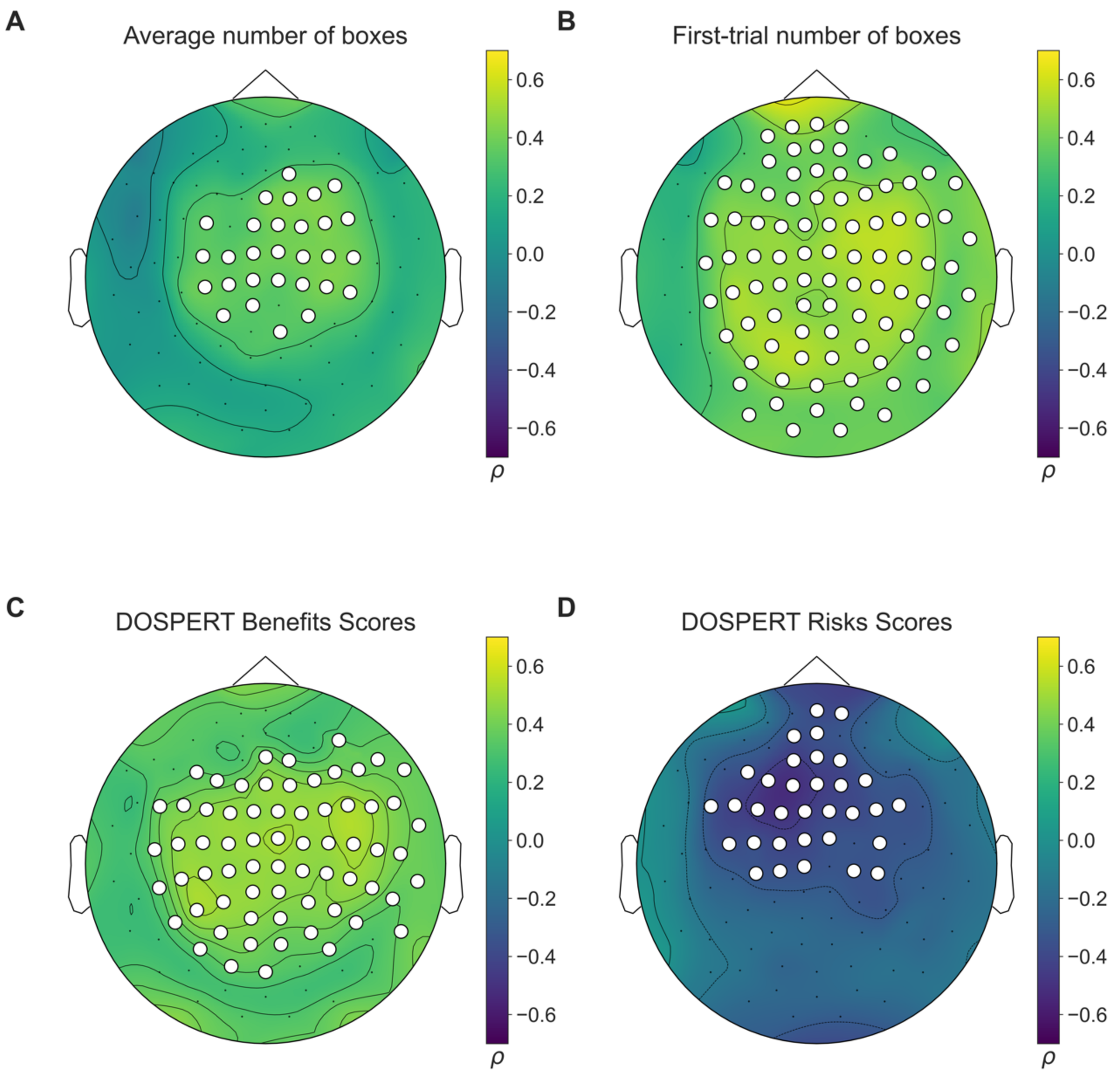

Supplementary Figure 4. Results of non-parametric statistical clustering in sensor space (1024 permutations) based on resting-state recordings before the game. Color denotes Spearman's correlation coefficients of theta power at sensor locations (102 magnetometers) with (A) average number of boxes chosen in the game; (B) number of boxes chosen in the first trial; (C) DOSPERT expected benefits subscale scores; and (D) DOSPERT perceived risks subscale scores. White dot markers denote magnetometers included in a statistically significant cluster (alpha level of 0.05). DOSPERT - Domain-Specific Risk-Taking Scale.

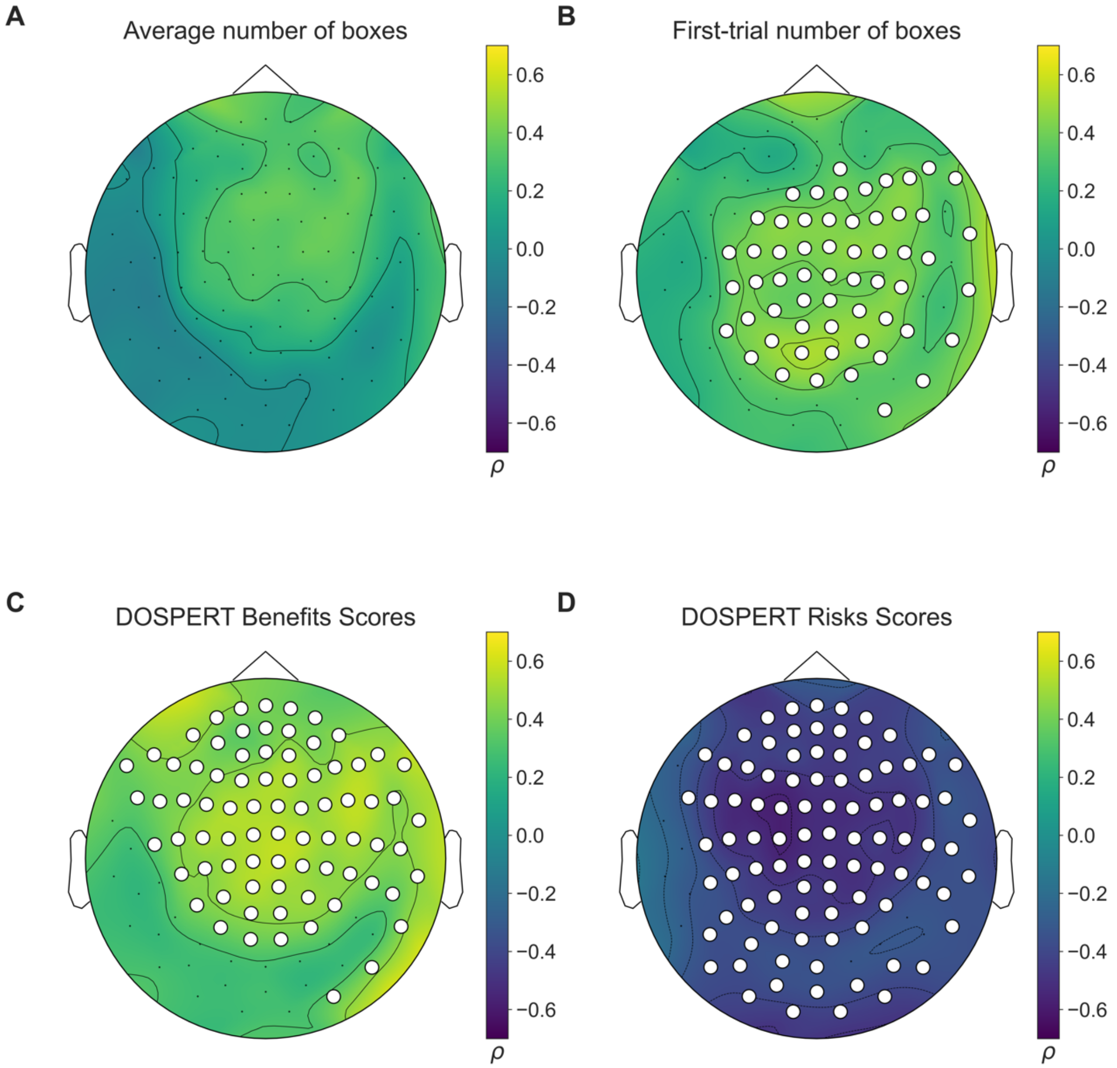

Supplementary Figure 5. Results of non-parametric statistical clustering in sensor space (1024 permutations) based on resting-state recordings after the game. Color denotes Spearman's correlation coefficients of theta power at sensor locations (102 magnetometers) with (A) average number of boxes chosen in the game; (B) number of boxes chosen in the first trial; (C) DOSPERT expected benefits subscale scores; and (D) DOSPERT perceived risks subscale scores. White dot markers denote magnetometers included in a statistically significant cluster (alpha level of 0.05). DOSPERT - Domain-Specific Risk-Taking Scale.

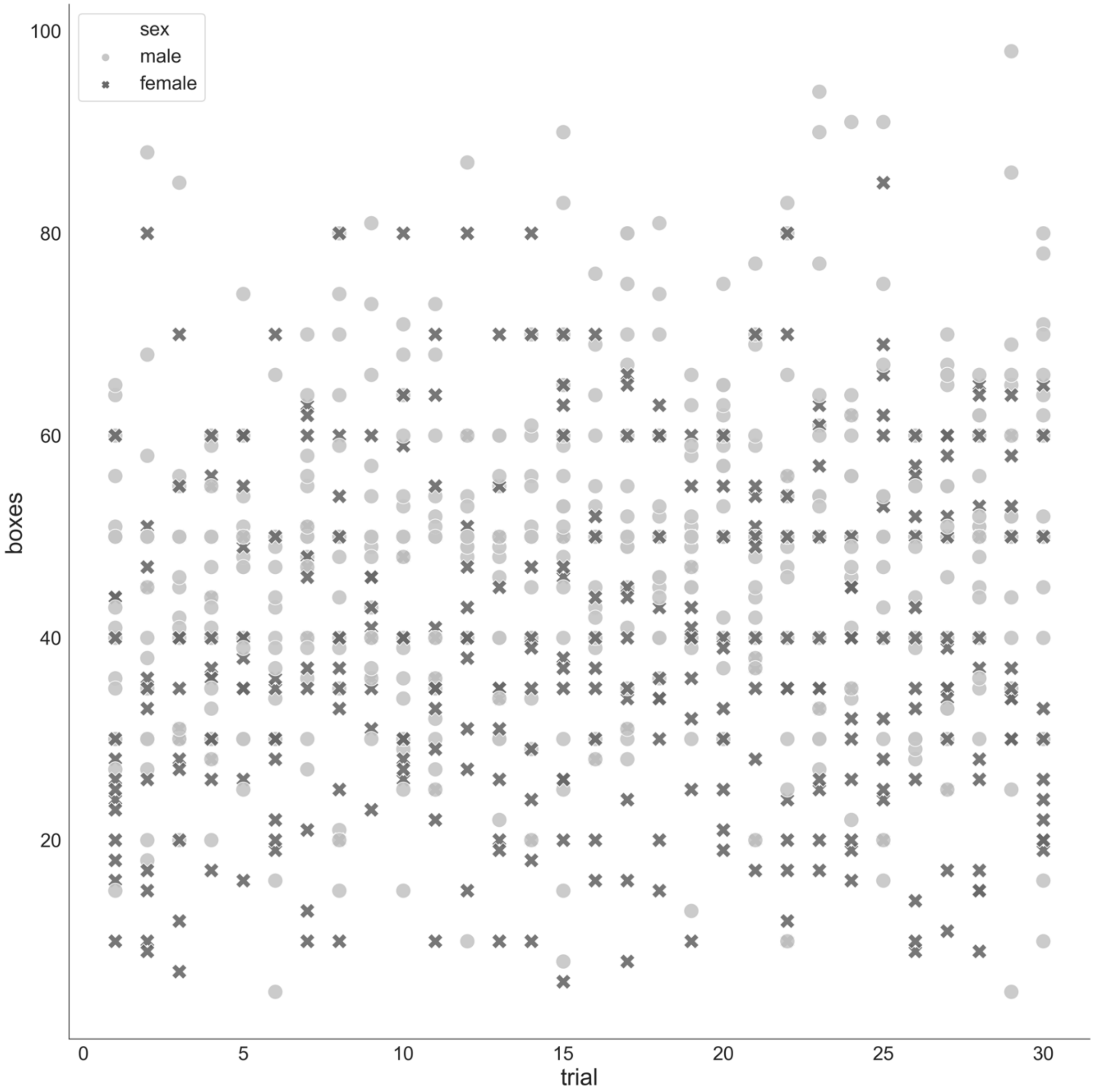

Supplementary Figure 6. Numbers of boxes chosen by each participant in each trial (n = 1041, trials with 0, 1 and 2 chosen boxes were excluded).

	rsFT	ACC theta	rsFT after game	ACC theta after game
% change after loss	0.33 (n.s.)	0.41 (0.01)	0.41 (0.02)	0.49 (0.003)
% change after win	-0.33 (n.s.)	-0.43 (0.01)	-0.29 (n.s.)	-0.47 (0.005)
Difference % change after win and loss	-0.37 (0.03)	-0.49 (0.002)	-0.36 (0.03)	-0.54 (0.001)

Supplementary Table 1. Spearman's correlation coefficients of rsFT/ACC theta power and average percent changes in the number of boxes after losing or winning. P-values for correlations significant after FDR-control are reported in parentheses.

Both average percent change after win (mean = 0.26; SD = 0.14) and percent change after loss (mean = -0.08; SD = 0.14) measures were statistically different from 0 (t-test p-values were 9.42e-07 and 0.001 respectively). We observed significant sex differences in percent changes in the numbers of boxes chosen after losing (Mann-Whitney U-test P = 0.007; mean males = -0.03; mean females = -0.15) and winning (Mann-Whitney U-test P = 0.01; mean males = 0.18; mean females = 0.36), and in the difference of these reactions (Mann-Whitney U-test P = 0.003; mean males = 0.21; mean females = 0.51).

	Dependent variable:							
	Average number of boxes				First-trial number of boxes			
	(1)	(2)	(3)	(4)	(5)	(6)	(7)	(8)
rsFTA before	41.19**	67.23***			20.01	45.84		
	(17.94)	(23.79)			(28.56)	(38.83)		
rsFTA after			20.43	74.77**			48.05	111.73**
			(21.91)	(33.02)			(30.66)	(47.22)
male	7.03**	4.82	7.84**	5.93*	12.55**	10.35*	11.76**	9.52**
	(2.99)	(3.22)	(3.20)	(3.17)	(4.76)	(5.26)	(4.48)	(4.53)
rsFTA before:male		−56.81				−56.35		
		(35.13)				(57.35)		
rsFTA after:male				−89.95**				−105.43*
				(42.48)				(60.76)
Constant	42.42***	43.83***	40.65***	41.91***	31.62***	33.02***	31.64***	33.12***
	(2.43)	(2.53)	(2.45)	(2.40)	(3.87)	(4.12)	(3.43)	(3.43)
Observations	35	35	34	34	35	35	34	34
R^2	0.29	0.34	0.18	0.29	0.21	0.23	0.24	0.31
Adjusted R^2	0.24	0.28	0.13	0.22	0.16	0.16	0.19	0.24
Note:						*p<0.1; **p<0.05; ***p<0.01		

Supplementary Table 2. Regression results used to assess interactions of rsFTA before and after the game and sex in relation to game-based risk-taking measures.

	Dependent variable:							
	Average number of boxes				First-trial number of boxes			
	(1)	(2)	(3)	(4)	(5)	(6)	(7)	(8)
ACC theta before	3.75**	3.16**			6.79***	6.51***		
	(1.60)	(1.52)			(2.32)	(2.37)		
rsFTA before		42.07**				19.83		
		(18.24)				(28.33)		
ACC theta after			4.15**	4.52***			4.84*	5.56**
			(1.60)	(1.59)			(2.40)	(2.32)
rsFTA after				31.81				62.54*
				(21.57)				(31.51)
Constant	44.86***	46.47***	44.61***	45.26***	38.03***	38.78***	37.24***	38.51***
	(1.58)	(1.64)	(1.58)	(1.61)	(2.28)	(2.54)	(2.36)	(2.35)
Observations	35	35	34	34	35	35	34	34
R^2	0.14	0.27	0.17	0.23	0.21	0.22	0.11	0.21
Adjusted R^2	0.12	0.22	0.15	0.18	0.18	0.17	0.09	0.16
Note:						*p<0.1; **p<0.05; ***p<0.01		

Supplementary Table 3. Regression results for linear models of risk taking on the two measures of neural characteristics.

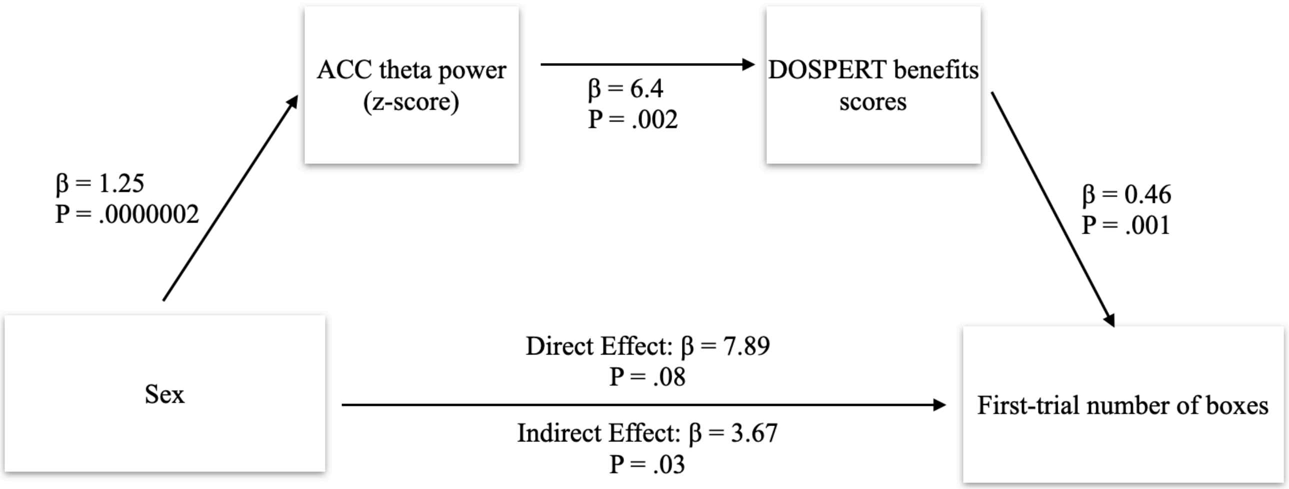

Supplementary Figure 7. Restricted structural equation model for sequential meditation of effects of sex on risk taking in the first trial of the game. β - regression coefficient; P - p-value of a regression coefficient. DOSPERT - Domain-Specific Risk-Taking Scale. ACC - anterior cingulate cortex.

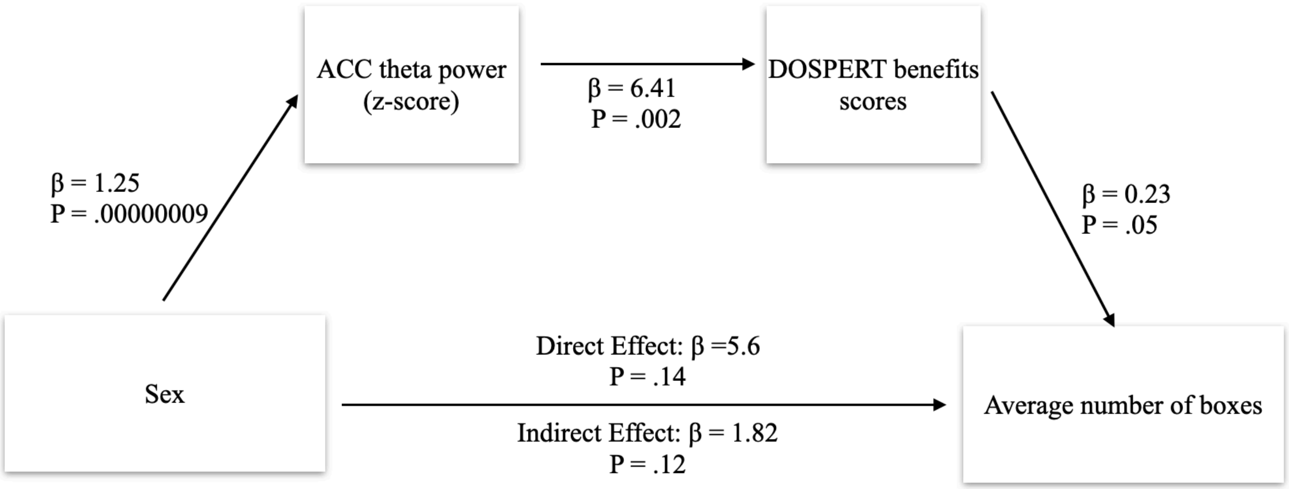

Supplementary Figure 8. Restricted structural equation model for sequential meditation of effects of sex on average risk taking in the game. β - regression coefficient; P - p-value of a regression coefficient. DOSPERT - Domain-Specific Risk-Taking Scale. ACC - anterior cingulate cortex.
